# Supplementary material for: AhR and CYP1B1 Control Oxygen Effects on Bone Marrow Progenitor Cells: The Enrichment of Multiple Olfactory Receptors as Potential Microbiome Sensors
Source: Int J Mol Sci. 2023 Nov 28;24(23):16884. doi: 10.3390/ijms242316884 (PMC10706615; doi:10.3390/ijms242316884)
Supplement: Supplementary file 1 [file ijms-24-16884-s001.zip › ijms-2663640-supplementary/ijms-2663640-supplementary.pdf]

Summary of Supplemental Figures

Figure S1. Adhesion effects on BMCs for males versus females, and C57BL/6J versus C57BL/6N.

Figure S2. Distribution of lymphoid and myeloid progenitor activities in Adherent (Adh) and non-adherent (NA) fractions. B-CFU: Pre-B progenitors; GM-CFU: Granulocyte-Macrophage Progenitors

Excel Files (Alphabetical)

A. Single mouse gene expression or 12 treatments (Cy3 Treatment/Cy5 NA reference).

B. Limma group comparisons with p values (all versus WT equivalent. *Cyp1b1*$^{-/-}$, *Cyp1a1*$^{-/-}$, Cyp1a1-BP, DKO, DKO-BP, BP 6 h, BP 12 h, BP 24 h, TCDD 12 h, DMBA 12 h (not *Cyp1b1*$^{-/-}$ DMBA vs *Cyp1b1*$^{-/-}$) Cyp1b1-DMBA.

Supplementary Tables

TableS1

A. Distribution of canonical (AhR/ARNT) versus BP-Cyp1a1 activations in Adh BM fractions. Change is presented as Limma comparisons of Treatment Group versus WT (Figure 2A).

B. Distribution of Treatment effects on 26 Cytokines, PG synthase/Ptgs2 and 16 Cytokine receptors in BM adherent fractions.

C. Selective effects of *Cyp1b1*$^{-/-}$ and *Cyp1a1*$^{-/-}$ on, respectively, lymphocytes and eosinophils.

D. Evidence for non-canonical AhR activation for *Cyp1b1*$^{-/-}$ AhR regulation.
DMBA effective inhibitor. BP activations effective through AhRd and AhRb alleles.

Table S2. Cyp1b1-AhR stimulations relative to WT basal (Limma statistics).

A. DKO(+) cluster genes, including DKO(+) NA sub-cluster/limma.

B. DKO(-)$^{-}$ cluster genes/limma.

C. DKO(+) relative expression levels.

D. DKO(-) relative expression levels.

Triplicate or duplicate set of gene Cy3/Cy5 (NA reference) ratios is averaged to provide a relative expression level that is further used for Limma comparisons shown in A and B.

Table S3. Application of Cyp1b1-AhR treatments to 44 OLFR genes.

A. Key treatment comparisons to WT with Limma statistics.

B. Division of OLFR between DKO(-) and constitutive expression.

Table S4. The specific role of A subunit or RNA Polymerase 2 in *Cyp1b1*$^{-/-}$ AhR activations.

Table S5. Matrix treatment responses show specificity at the level of individual mice that confirms mechanism retention.

Table S6. Suppression through Cyp1b1-AhR treatments.
Focus on lymphocytes, erythroblasts, and osteoblasts.

Table S7. Overlap between adherent BMCs for expression of OLFR.
A. BMS2 cells share many DKO(+) genes that are expressed in adherent BMCs.
B. Expression of 10 OLFR in basal WT BM cells and BMS2 cells.

Table S8. Comparison of OLFR genes expressed in BM cells with reported expression in Pancreatic Islets and MIN cells.

Table S9. Primers used for RT-PCR analyses of BM macrophages.

Supplementary Text addressing analysis of CYP1-AhR partnerships, Polr2a and OLFR expression (Figures 2 and 3 with Tables S1-S8).

1. Introduction to supplement sections

Our previous work has resolved two mechanisms through which PAH affects genes in BM cells via activation of the AhR/ARNT complex [1]. However, the present work shows that most of the stimulations function through a process that is closely replicated by the effects of Cyp1b1 deletion, which closely replicates the effects of TCDD activation of AhR and, more effectively, of BP effects in *Cyp1a1*$^{-/-}$ mice. These processes are highly influenced by the surface adhesion of freshly isolated BM cells (Figure 2B, Table S1A, S1B). A new perspective on AhR activity in BM cells has emerged that was totally unanticipated in the original study plan (Figure 2A).

Critically, no more than 5 percent of freshly isolated BM cells engage in this cell adhesion, but multiple cell types participate. Cyp1b1 is expressed in multiple BM cells, and we have previously shown that both Cyp1b1 and TCDD extensively redirect cell adhesion [1, 2] (Figures 1A, 1B, 1D). These experiments expand our previous evidence that Cyp1b1 is a cell-selective modulator of redox signaling associated with DNA double strand break repair (DDSB)

(Lymphoid lineage, Figure 4), NF-κB (Endothelial cells, Figure 5) and Pparɣ (Myeloid progenitors, Figures 6, 7) [3, 4]. The original goal of demonstrating cell selectivity is presented through generation of *Cyp1b1*$^{fl/fl}$ and lineage selective Cre deletions.

The gene expression data shown in two excel files (Supplemental excel files A and B), are comprised of responses from 12 treatments that are introduced in Figure 2A. Supplemental Tables S1-S7 provide comprehensive data from over 200 genes. These 12 treatments systematically engage different features of AhR activity and reveal the very distinctive contributions of Cyp1a1 and Cyp1b1. These tables also emphasize the precision that is available when dual Cy3/Cy5 labeling is applied to microarray analyses. Cyp1b1 experiments with RNAseq provide a different type of data on similar networks [5]. The detailed discussion of the Supplemental Tables, provided here, focuses on a pharmacological approach to the resolution of signaling, based on this matrix of treatments. The primary text introduces novel gene changes that have been identified through dual responses to Cyp1b1 and AhR (OLFR forms and Polr2a). This supplement provides a more detailed analysis of the multiple OLFR forms and of Polr2a among the complete 12 subunits.

2. BM cell isolation and selective presence of endogenous AhR ligands in adherent cell assemblies

The cell content of the adherent cell assembly changes after in vivo treatment, usually coupled to the intrinsic gene expression of the cells. In the basal WT state, there are only modest differences between adherent and non-adherent cells. Few genes show expression differences of over 2-fold between the adherent and non-adherent fractions (WT adhesion ratio; Adh/NA). The present studies have used RNA from basal WT non-adherent cells as a reference standard (Cy5) for the dual labeling of the arrays. The Cy3 expression for each treatment sample is expressed as a ratio to the same Cy5 NA reference sample. Individual Cy3/Cy5 ratios for each treatment group (usually n=3) were analyzed using the Limma-Edge algorithm. Treatment effects are presented as a ratio of the average of the 3 treatment samples to the average of the adherent WT BM cells (Figure 2, Tables S1A, S2A, S2B).

The cells were isolated within 60 min, thus maintaining the in vivo gene expression patterns for all but the most labile mRNA. The cell isolations have been completed under ambient oxygen levels, which are far above levels in the BM sinusoids. The impact of this change for even 60 min on labile mRNA is notable for cytokines and early response genes (Tables S1A, S1B). This point is particularly important since we show, here, that Cyp1b1 controls the effects of elevated oxygen over the course of 24 h.

3. Design for Different in vivo Treatments prior to BM cell isolations

Direct AhR activation was compared for 12 h intra-peritoneal (IP) treatments with TCDD, BP or DMBA. TCDD, unlike the PAHs, does not generate reactive metabolites, but effectively demonstrates the effects of AhR activation in BM [6]. WT mice were treated with BP, via IP injection, for 6 h, 12 h and 24 h, thus extending well beyond the major blood clearance time. This is complete after 6 h, apart from a small proportion that exhibits slow release from abdominal fat [7].

The administration of PAH to *Cyp1a1*$^{-/-}$ (or DKO) mice slows their hepatic clearance relative to WT mice, while lowering the reactive metabolites. Cyp1b1 is scarcely expressed in hepatocytes but dominates non-bacterial contributions to AhR activation in gut epithelia [8] or basal expression in BM vascular cell types and MSC that line the sinusoids. Additionally, Cyp1b1 has a more extensive basal extra-hepatic presence. Deletion has only local effects on metabolism. Greater effects on local reactive metabolites have been established for BM.

4. Canonical AhR responses are a minority

Canonical AhR responses with ARNT pairing show similar BP responses in WT mice at 6, 12 and 24 h [1]. This stimulation is maintained in *Cyp1*$^{-/-}$ mice and may be elevated due to more sustained presence of BP (Figure 2B, Table S1A). The comparisons for the most responsive canonical markers, Ahrr and Spint1/Hai1, are shown for 30 mice across all treatment groups in Table S5. The parallel responses for individual mice are striking. There are only eleven genes showing this pattern, each with very low expression in the adherent assemblies (Figure 2E). Low TCDD responses relative to BP (Cyp1b1, Dpep2, Gpnmb) are attributable to the elevated retention in abdominal fat. Two AhR responses (Cxcl2, Cxcl3) show a mixed canonical response, typified by a TCDD stimulation that is supplemented by a response to BP metabolites. *Cyp1a1*$^{-/-}$ responses are significantly increased, demonstrating a peak response to BP at 6 h that disappears at 24 h. This pattern is indicative of a canonical response supplemented by a labile 6 h response to a BP metabolite that is not generated in *Cyp1a1*$^{-/-}$ cells.

Tiparp and Nqo1 are typical canonical markers [6, 8-10] that exhibit much higher expression than the BM canonical AhR set, but only small BP-induced AhR responses that are maintained for 12 h. Their primary expression is probably in different cell types in which AhR activity is low. The BP stimulation is maintained for 24 h but, like Cyp1b1, is not reproduced by TCDD.

5. BP-Cyp1a1 stimulation

BP is converted by Cyp1 metabolism to two types of reactive metabolite; BP dihydrodiol epoxides (PAHDE) and primary BP quinones (3, 6; 1,6 and 6,12). DMBA more effectively forms PAHDE but cannot form primary quinones [1, 7]. PAHDE are highly electrophilic and potently form quantifiable DNA adducts that lead to DDSB. These DNA changes lead to cell signaling changes within a few hours; notably ATM kinase and p53 activation [11, 12]. BP quinones, at low levels, undergo redox cycling to generate oxygen radicals, hydrogen peroxide and lipid peroxidation products, collectively called ROS. Cyp1b1 more effectively generates PAHDE than Cyp1a1, which has higher selectivity for quinones.

The BP-Cyp1a1 stimulation, which extends to 17 genes (Figure 2D, Table S1A) depends on BP metabolism to a BP primary quinones. Stimulation of these genes is absent in *Cyp1a1*$^{-/-}$ mice, thus implicating Cyp1a1-mediated BP metabolism as a necessary step. For Ptgs2, IL1b, Ccl3, Ccl4, Cd14 and Egr1, stimulation by TCDD is absent, thus excluding direct AhR activation from this process. These genes are no longer enhanced when the normal AhRb allele is replaced by the AhRd allele, which does not bind PAHs [9, 13]. The response is at a peak within 6 h and then declines rapidly within 12 h. This time course can only be met by labile mRNA (t1/2<1 h), which is typical of cytokines and many transcription factors.

Most of the BP-Cyp1a1 genes also show a TCDD stimulation coupled to an increase in *Cyp1b1*$^{-/-}$ mice (Figure 2D, Table S2A, S2B). This pairing is the signature of the Cyp1b1-AhR partnership, which we discuss below. This mechanism also functions through ROS, likely functioning through different cells.

A more complete list of 26 cytokines expressed in the BM cells (Table S1B) shows that 11 genes match Ptgs2 in the BP-Cyp1a1 response. The *Cyp1b1*$^{-/-}$ overlaps with *Cyp1a1*$^{-/-}$ BP in Table S1B applies to IL10, IL2, IL15, IL27, Ccl2, Ccl19, Ifnα2, IL6 and Cxcl10. Each is also accompanied by smaller TCDD stimulation. This pattern is the signature of the Cyp1b1-AhR process. Among receptors, only IL17rd shows any of these regulatory steps functioning at the level of mRNA expression.

6. Additional selective Cyp1 deletion mechanisms revealed

Several other different activation patterns have been recognized from this multi-treatment approach. B cells that synthesize heavy chain immunoglobulins (IgH) are selectively targeted by *Cyp1b1*$^{-/-}$ and, to a lesser extent, DKO (Table S1C). Direct activation by TCDD has no impact. BP, however, is active, probably through the BP-Cyp1a1 mechanism. The IgH genes are processed by a natural rearrangement of DDSB [14]. We suspect that IgH are particularly

sensitive to ROS perturbation derived from either *Cyp1b1*$^{-/-}$ or BP-Cyp1a1 processes. The sensitivity of lymphoid progenitors to ROS is addressed in Figure 4.

Tnf is distinguished from Ptgs2 and IL1β through stimulations in *Cyp1a1*$^{-/-}$ and DKO cells (Figure 2B, Tables S1B, S5) and by TCDD activation. Hspa1a (Hsp70) shares response features, notably large increases in both *Cyp1a1*$^{-/-}$ cells and to direct WT activation by TCDD (12 h). The *Cyp1a1*$^{-/-}$ increase may reflect activation by a constitutive AhR activator from the gut that is metabolized by Cyp1a1 [15, 16]. This *Cyp1a1*$^{-/-}$/TCDD selectivity is also seen in eosinophils with suppression of highly expressed EAR (Eosinophil activated Ribonucleases; EAR 3, 6, 7 and 10) (Table S1C) that are released from eosinophil particles along with the peroxidase, Epx. The eosinophil marker peroxidase, Prg2, is unaffected, indicating that the particles rather than the cells are depleted. Eosinophils are evidently a major component of the assembly.

7. Cyp1b1-mediated suppressions

Suppression patterns derive substantially from adherent cell losses associated with *Cyp1b1*$^{-/-}$ cells and TCDD treatment. The stimulations of IgH, attributed to a subset of B cells, occur with 3-fold losses. In Figure 2C, the Volcano plot shows a narrow band of *Cyp1b1*$^{-/-}$ suppressions marked by Rag1, a mediator of B cell immunoglobulin processing. This band corresponds to genes expressed in cells that dissociate from the adherent assemblages, including B- lymphocytes. However, Cd79b, the most highly expressed of the B cell markers, exhibits high *Cyp1b1*$^{-/-}$ suppression without TCDD and BP effects. Another highly expressed B cell marker, Lgals3/Galectin3, has a similar profile (Table S6).

Distinct B cell subsets are shown by markers with lower expression. Ms4a1/Cd20 and Vpreb3 share the Rag1 response pattern (Figure 2D-right) in which the *Cyp1b1*$^{-/-}$ suppressions are matched by TCDD and BP WT treatments. A further group of suppressed genes (Klhl6, Fcrla and Cbfa2t3) repeat the Cyp1b1/TCDD pairing but fail to respond to BP (Table S6). Functional resolution of different B cell types is a feature of immune responses.

Erythroblasts are regulated by AhR [17]. Late erythroblasts, marked by highly expressed Epb4.1, exhibit a distinctive preference for adherence. These genes show *Cyp1b1*$^{-/-}$ suppressions with parallel TCDD decreases. This cluster is distinguished from B cell clusters by absence of DKO decreases. Hemoglobin genes that are expressed highly in erythroblasts lack adherence preference and show stimulation increases from *Cyp1b1*$^{-/-}$ cells or TCDD treatment. These stimulations could arise from a decreased progression of these erythroblasts. Figure 2D

and Table S6 also shows decreases in the progression of osteoblasts marked by Collagen genes.

8. Cyp1b1-AhR Partnership divides into two clusters based on DKO responses

The losses that appear in the Volcano plot in Figure 2C as a narrow band from -2 to -4 are matched by similarly restricted stimulations of over 200 genes by TCDD (Tables S2A, S2B). The *Cyp1b1*$^{-/-}$ stimulations expand the plot through superimposed intrinsic stimulations (Figure 2D). The group resolves into DKO(+) and DKO(-) clusters based on differences in adhesion of WT BM cells and different stimulatory effects of *Cyp1a1*$^{-/-}$ that are lost in DKO cells. The largest cluster comprises 75 genes, including 21 of the OLFR genes ([DKO(+)] (Tables S2A, S3B). A further 12 highly responsive gene [DKO(+)^{NA}] stimulations lack WT adherence suppression. No OLFR genes are included in this sub-cluster. In the DKO(+)^{NA} group, the low adhesion effects remove this variance to clarify the significance of the TCDD and BP stimulations and the lesser effects of *Cyp1a1*$^{-/-}$ (Table S2A).

The smaller DKO(-) cluster also includes OLFR genes. Their rarity suggests that the two clusters derive from a common cell type in which adhesion signaling couples asymmetrically to genes in the two clusters. The DKO(+) genes distributed to much higher abundance than the DKO(-) genes. Thus, 20 percent exceed an expression level of 10,000 Cy3 units with 28/52 exceeding the highest expression DKO(-) genes (3400) (Figure 3C, Table S2C, S2D).

The WT DKO(+) gene suppressions have appreciable diversity. TCDD and BP treatments and the switch to *Cyp1a1*$^{-/-}$ essentially remove this suppression, whereas DMBA maintains the suppression. In DKO(+) genes, the diversity of adherence differences means that the *Cyp1a1*$^{-/-}$ stimulations are not significant. However, the low adherence effect exposes an appreciable, significant *Cyp1a1*$^{-/-}$ stimulation. Unlike canonical genes, there is a robust stimulation that may correspond to Cyp1a1 substrates derived from the gut microbiome [8, 18]. For Cyp1b1-AhR genes, the transition from BP in WT cells to BP in *Cyp1a1*$^{-/-}$ BM cells exceed the canonical response genes, such as Ahrr or Spint1. This weaker ligand binding then requires the extra systemic BP provided by deletion of liver Cyp1a1 (BP-*Cyp1a1*$^{-/-}$ stimulations). In the WT mice, there is a mean 3.5-fold suppression across BM cells.

TCDD and BP treatments and the switch to *Cyp1a1*$^{-/-}$ essentially remove this suppression, whereas DMBA maintains the suppression. However, DKO(-) genes lack this confusion, such that *Cyp1a1*$^{-/-}$ stimulation are about 25 percent of the *Cyp1b1*$^{-/-}$ stimulations. These increases then provide evidence for endogenous ligands that attain 25 percent of maximum stimulation. We are proposing that Cyp1b1 deletion effects are more effective

because of the elevation of ROS that provides additional AhR stimulation through alternative partnering, such as from RelA [19, 20].

The effective suppressions of *Cyp1b1*$^{-/-}$ stimulations by DMBA indicates antagonist activity. In the same cells DMBA induces Ahrr and Spint1 comparably to BP (Table S1D). This failure to activate AhR in all DKO(+) and DKO(-) genes is indicative of a non-canonical partnership [19, 20]. Table S1D also shows that BP can activate these genes as effectively as the normal TCDD induction with the normal AhRb allele of C57BL/6J mice.

9. A third of these stimulations are OLFR Genes. Match to Islet cells

The retention of multiple OLFR and Defensin 3 gene expressions in BMS2 cells suggests a close relationship of the DKO(+) and DKO(-) genes to this line, which also derives from the BM-MSC (Tables S7A, S7B). Over 30 genes from the olfactory receptor GPCR family (Figure 3D, Tables S3A, S3B) correspond to 1000 olfactory genes that are expressed in the neurons of the olfactory bulb [21]. There, they respond cooperatively to small volatile chemicals. We identified the highly expressed Olfr1410 in the Volcano plot (Figure 2C). The alphabetical listing of a near complete set on the microarray revealed a further 40 OLFR genes (Tables S3A, S3B). Nearly all genes that were expressed exhibited the pattern of Cyp1b1-AhR stimulations. Six OLFR genes (Table S3B) share robust expression but low responses to *Cyp1b1*$^{-/-}$ and AhR activations. These forms, therefore, exhibit constitutive expression with normal Cyp1b1 and AhR expression.

Only Olfr571 exceeded 10,000 Cy3 unites, while the most responsive, Olfr1410, exceeded 5000 and a further ten exceeded 1000. Only 1/34 OLFR with expression >300 failed to show two-fold stimulation by *Cyp1b1*$^{-/-}$ (Tables S7A, S7B).

In Table S8, we show that there is appreciable overlap between 22 OLFR expressed in BM cells and 48 identified in Islet cells or the MIN cell line. The BM OLFR are, with two exceptions, stimulated by Cyp1b1$^{-/-}$ and *Cyp1a1*$^{-/-}$ BP. We consider that in each tissue the olfactory receptor is re-purposed to provide a response to circulating microbiome products. Octanoic acid, which represents a typical microbiome short chain fatty acid product, enhances insulin release [22]. In the BM cells they might enhance the expansion of BM-MSC marked by DKO(+) genes or release Defensin 3.

10. The specific role of A subunit of RNA Polymerase 2 following stimulation by *Cyp1b1$^{-/-}$*. Correlation with multipotency marker, H1fx

The highest stimulation is provided by **the Polr2a gene**, which encodes the catalytic A subunit of RNA polymerase A. There are, however, 11 other subunits that do not change their expression as Polra2a engages in a 30-fold increase (Figure 3F, Table S4) [23, 24].

Polr2a expression establishes the pattern for all other DKO(+) genes. Thus, *Cyp1b1$^{-/-}$* BM and *Cyp1a1$^{-/-}$* BP each change the 3-fold suppression into a 10-fold stimulation relative to non-adherent cells. The DKO mice, with or without BP, produce a lower, but substantial 3-fold elevation.

The relative expression levels of the 12 subunits across adhesion and *Cyp1b1$^{-/-}$* changes are shown in Figure 3F. Subunits E, G and L are always the highest, including in BMS2 cells, while H is the lowest. The peak stimulation by *Cyp1b1$^{-/-}$* brings the A subunit up to the B subunit level. The treatment effects on the Polr2 subunits are compared in Table S4. The low expression H subunit shows a trend to suppression by *Cyp1a1$^{-/-}$.* The function of these subunits is complex and rarely documented. It seems likely that specific interactions with subunits control the location, release, and specificity of RNA polymerase 2. For example, XPA interacts with Polr2 to couple RNA transcription to DDSB.

The core mechanism for control of DKO(+) genes by Polr2a involves an activation by the Cyp1b1-AhR mechanism that overcomes basal adhesion repression. The extended presence and rare expression of OLFR suggest that DKO^{-} genes are expressed in this same cell type through Polr2a, but with distinguishing linkages to Adh and Cyp1a1.

Polr2a is also associated with very high Cyp1b1-AhR stimulation of the linker histone, H1fx (Tables S2A, S2C). The expression is the highest in the DKO(+) cluster and completely dominant over locus partner, H1f0. In mouse embryos, H1fx is co-expressed with Nestin during organogenesis [25]. This linker histone maintains chromatin in a pluripotent state [25]. The individual mouse expression for H1fx is compared in Table S5.

This expression level of H1fx, however, is over three times the expression of Chd4 and Defcr3 (30,000-33000), the highest DKO(+) genes, even though retaining the preferred expression in the N-Adh fraction. The exceptionally high expression levels suggests that H1Fx is also expressed in an additional abundant cell-type that does not share the DKO(+) characteristics. For example, erythroblasts express hemoglobin (Hbb-b1) at levels comparable to H1Fx that are also stimulated by *Cyp1b1$^{-/-}$*, although without adhesion sensitivity (Table S6). The H1Fx expression pattern could represent a mix of BM-BMSC and erythroblast.

Supplement Figures:

Figure S1

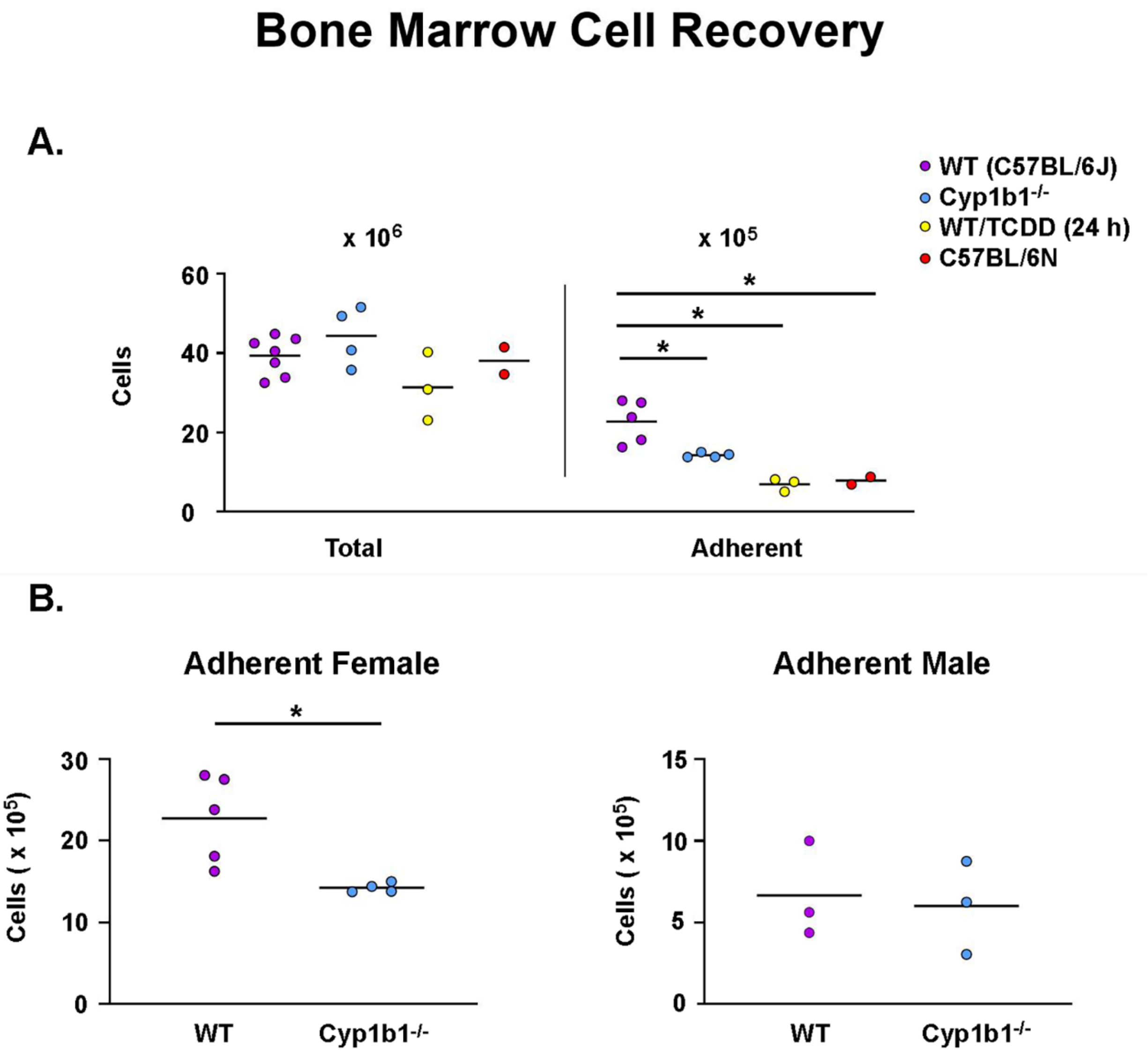

Figure S2

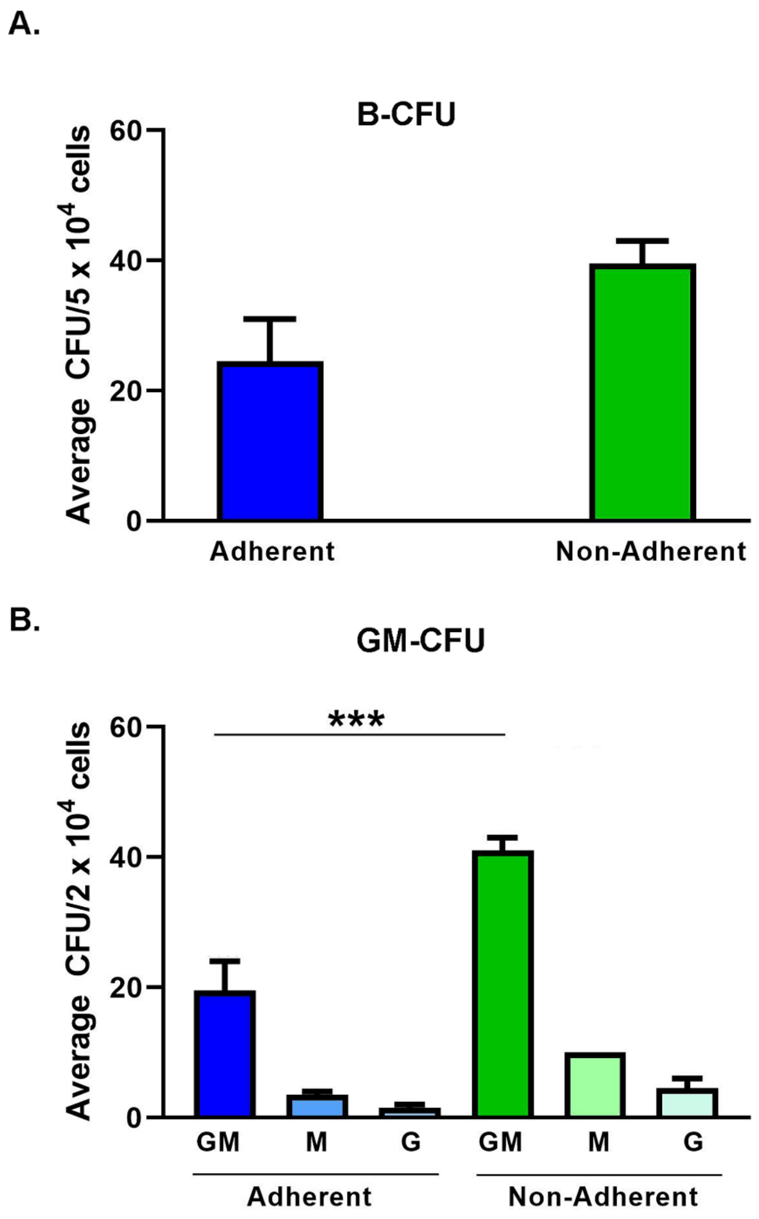
A.
B-CFU
Average CFU/5 x 10^4 cells
60
40
20
0
Adherent
Non-Adherent
B.
GM-CFU
Average CFU/2 x 10^4 cells
***
60
40
20
0
GM
M
G
GM
M
G
Adherent
Non-Adherent

Supplement Tables S1-S8

Table S1A. Distribution of canonical (AhR/ARNT) versus BP-Cyp1a1 activations in adherent (Adh) BM fractions.

	$1b1^{-/-}$	$1a1^{-/-}$	BP-$1a1^{-/-}$	DKO	DKO-BP	WT-TCDD	WT-BP 6	WT-BP 12	WT-BP 24	AhRd BP 12
**Reference**	**WT**	**WT**	**1a1KO**	**WT**	**DKO**	**WT**	**WT**	**WT**	**WT**	**WT**
**AhR/ARNT**										
**AhRR**	nc	nc	**10.4****	nc	**23.0****	**3.8****	**8.3****	**2.7**	**7.3****	**-2.4**
**Spint1**	nc	nc	**12.5****	nc	**27.9****	**5.0****	**5.2****	**4.1****	**6.3****	**-10**
**Gpnmb**	nc	nc	**9.0****	nc	14.8*	**nc**	3.5*	**2.1**	3.5*	**-4**
**Acpp**	1.6	nc	**3.7****	nc	3.5	**3.5****	**3.4****	**4.0**	**3.8****	**-1.9**
Cyp1a1	nc	----	----	----	----	**10.0**	**11.4**	**9.5***	9.8*	**-7.6**
**Cyp1b1**	---	nc	**3.0****	----	----	nc	**3.3****	**2.4**	**4.5****	**-2.3**
**Hic1**	nc	nc	2.4	nc	5.7*	**4.1****	**8.4****	**5.5****	**5.9****	**-1.8**
**Dpep2**	nc	nc	**4.2****	nc	2.4	**nc**	**3.0****	**2.1**	**2.4****	**-1.5**
**Dnahc2**	1.6	nc	**3.5****	nc	**3.2****	**2.2****	**4.6****	**nc**	**2.7****	**nc**
Cxcl3	nc	nc	4.9*	nc	5.4	3.7*	10.4**	5.0**	nc	**-4**
**Cxcl2**	nc	nc	1.7	nc	2.5	**4.2****	**14.0****	11.2	**6.5****	**nc**
Tiparp	nc	**nc**	Nc	nc	nc	nc	**2.5****	2.3**	**1.5****	**nc**
Nqo1	nc	nc	Nc	nc	nc	nc	1.6**	1.6**	2.7**	**nc**
**BP-Cyp1a1**										
**Ccl3**	1.7	nc	Nc	**1.7**	nc	**1.5**	**2.8****	2.8**	-2.6**	**-3.2**
**Ccl4**	1.3	nc	Nc	**1.9**	nc	**nc**	**3.9****	1.9**	-2.3**	**nc**
Il1b	1.4	nc	Nc	nc	nc	nc	**4.4****	2.5**	-4.0*	**-1.6**
Ptgs2	-2.3	**nc**	Nc	nc	nc	nc	**10.0****	2.7**	-1.6	**-2.4**
**Ifng**	**3.2****	nc	Nc	4.2*	nc	**2.5***	**3.1****	1.3	-1.3*	**-1.7**
**Il10**	3.5**	nc	**2.4****	4.5*	nc	**2.9***	**4.7****	1.7	**1.7***	**nc**
**Il6**	**2.8****	2.6*	Nc	**6.0****	-1.5	**3.0****	**4.7****	1.5	**-4.2****	**-1.3**
**Cxcl10**	1.5	1.7**	nc	2.4*	nc	**2.0****	**7.6****	1.8	**-1.8****	**-2.8**
**Tnf**	1.3	2.9**	Nc	**3.4****	nc	**2.0****	**5.5****	3.3	-1.4*	**-1.4**
**Il1a**	1.6	nc	Nc	4.7*	nc	**5.5****	**9.2****	3.1	1.4	**-2.5**
**Cd14**	-1.6	-2.0*	1.8	-1.6	1.3	nc	**3.0****	1.9	**nc**	**-1.5**
**Cd69**	nc	nc	Nc	**2.7****	nc	**5.2****	**5.6****	2.5**	nc	**nc**
**Nfkbiz**	nc	1.7	Nc	**2.5****	nc	**2.9****	**9.3****	4.6**	nc	**1.4**
**Egr1**	**2.0****	2.0	Nc	nc	nc	**nc**	**5.4****	6.4**	nc	**-6.9**
**Egr2**	1.5*	1.7	Nc	**1.6**	nc	**2.9****	**5.9****	1.9	nc	**-2.5***
Maf f	**2.1****	1.8*	Nc	**2.0**	nc	**2.4****	**2.7****	2.3**	nc	**nc**
Socs3	1.9**	1.6**	Nc	1.6*	nc	**1.7****	**2.6****	1.5*	nc	**-2.2***

Table S1B. Distribution of treatment effects on 26 cytokines.

Gene Cyt	Exp 10^{-3}	BP6 WT	BP24 WT	TCDD WT	WT Adh/NA	1b1$^{-/-}$ WT	1a1$^{-/-}$ BP	Gene CytR	Exp 10^{-3}	1b1$^{-/-}$ WT	1a1$^{-/--}$ BP
ccl2	2	2	-1.5	1.3	1	1.6	1.8*	ccr2	2	nc	-1.6
ccl3	7	4.0*	-2	nc	1	2.0	nc	ccr5	1.5	-1.5**	nc
ccl4	4	4.0*	-2	nc	1	1.5	nc	ccr6	1	nc	nc
ccl5	10	nc	nc	nc	-2	nc	nc	ccr7	4	-1.7*	nc
ccl6	25	nc	nc	nc	-1.6	nc	nc	ccr9	1.5	-1.8	nc
ccl9	4	nc	nc	-2	1	nc	nc	cxcr4	6	1.5*	1.8
ccl19	1	nc	nc	2.0	1	8.0*	3.4*	cxcr5	3	-2.8**	nc
ccl25	1	nc	nc	nc	1	1.7	nc	il10ra	2	-1.5	nc
cxcl10	1	9.0	-2	2.0	1	1.7	2.1*	il10rb	8	-1.7	nc
cxcl2	0.2	25	-3	8.0	2.0	nc	2.2	il11ra	3.5	2.2**	nc
cxcl3	0.1	14	2	5.0	1.5	-2.5	3.2	il17ra	2	nc	nc
cxcl12	1.5	-1.5	nc	-4	-1.5	2	nc	il17rd	2	5.5**	4.5*
il1b	9	5.0*	-4.5	nc	1.5	1.5	nc	il1r1	0.5	nc	nc
il10	.05	4.0	1.5	1.5	-1.5**	4.0*	4.1*	il1r2	7	nc	nc
il12a	1	1.6	nc	-1.5	nc	-1.5	nc	ifngr1	40	nc	nc
il15	0.3	nc	nc	2	-1.6**	5.0*	3.4	ifngr2	5	nc	nc
il16	2.5	-1.5	nc	-1.5	1	nc	nc				
il18	0.4	nc	nc	nc	-1.3	2	1.5				
il2	0.07	nc	nc	2.0	-1.5	2.6*	2.7*				
il4	0.7	nc	-2	nc	-1.5	nc	nc				
il6	0.5	5.0*	-4	3.0	nc	3.0*	2.6*				
il27	1.0	nc	1.5	2.3*	-1.4	2.8*	2.6*				
il33	0.1	nc	-5	nc	-1.5	nc	nc				
Ifng	0.1	2.3	-1.7	1.8	-1.5	nc	nc				
ifna2	0.1	3	nc	1.6	-1.7**	5.0*	5.2*				
Tnf	0.7	8	nc	2.8	1.3	nc	(2.0)				
Ptgs2	0.1	10	nc	nc	2.8	nc	nc				

Table S1C. Cell Type Selective *Cyp1b1*$^{-/-}$ and *Cyp1a1*$^{-/-}$ effects.

**Adherent Ratio Gene**	**WT Adh/NA**	**Cyp1a1$^{-/-}$ WT**	**Cyp1b1$^{-/-}$ WT**	**DKO WT**	**TCDD WT**	**BP-12h**
**Cyp1b1 stress**	**B Cells**					
Igh-2	nc	2.4**	**6.8****	**3.7****	nc	1.9**
Igh-Vj	nc	1.5	**4.7****	**2.3**	nc	1.7**
Igj	nc	1.8	**3.7****	**2.3****	nc	2.4**
**Cyp1a1-TCDD**	**Eosinophils**					
Hspa1a	nc	5.4**	**2.6**	**2.9**	**8.6****	nc
Hspa1b	nc	5.7**	**2.2**	**2.3**	**5.8****	nc
Tnf	1.3	2.9**	1.3	**2.3**	**2.0****	**3.0****
Ear1	-1.7	nc	nc	**nc**	**-2.0****	nc
Ear6	-1.4	**-2.7****	**-1.4**	**nc**	**-2.4****	nc
Ear10	-1.35	**-2.3****	nc	**nc**	**-2.2****	nc
Epx	nc	**-1.9****	**-1.9**	**-1.4**	**-2.3****	nc
Prg2	nc	**nc**	nc	**-1.4**	**nc**	nc

Table S1D. Non-canonical effects on adherent content (DMBA, BP_{AhRd}).

Adh Fractions<br>A/B	WT<br><br>Adh/NA	$1b1^{-/-}$<br><br>WT	$1b1^{-/}$DMBA<br><br>$1b1^{-/-}$	TCDD Ahrb<br><br>WT	BP Ahrd<br><br>WT
**Gene**					
**AhR/ARNT**					
**Ahrr**	**2.8**	-2.2	**10.0***	3.8**	**-2.5**[a]
Spint-1	**1.7**	-2.5	**11.6****	5.0**	**-10.0**[a]
**DKO(+)**					
**Polr2a**	-3.4	28.1**	**-3.5****	3.5*	**4.7***
**Nes**	-2.8	7.8**	**-3.1****	3.7*	**3.8***
**Prp2**	-2.1	12.1**	**-3.5****	3.4**	**3.2***
Shc2	-2.5	13.2**	**-3.1****	3.7*	**3.8***
**Chd4**	**-1.8**	8.1**	**-2.6****	3.0**	**3.9****
**Pip5k1c**	-1.9	8.8**	**-3.1****	3.3**	**3.0****
**DKO(-)**					
**Chrm4**	**-1.2**	**12.8****	**-4.9****	**1.9***	**4.5****
**Ccl19**	**-1.3**	**9.9****	**-4.7****	**2.9****	**2.5***

Table S2. Cyp1b1-AhR stimulations relative to WT basal (Limma statistics).

Table S2A. DKO(+) cluster.

Gene	**1b1$^{-/-}$**	**1a1$^{-/-}$**	1a1$^{-/-}$ BP	**DKO**	**WT TCDD**	WT BP	WT Adh
**STIMULATION Fold Change**	**WT**	**WT**	1a1$^{-/-}$	**WT**	**WT**	WT	WT NA
*Limma p***				**DKOBP/DKO nc**			
**Kcnh4**	**30.7****	**3.2**	4.9	**14.6***	**3.5**	4.2*	-3.7
**Polr2a**	**28.0****	**3.1***	5.0	**11.1****	**3.6***	4.8**	-3.5
**Ugt2b34**	**25.7****	**3.0***	5.2	**7.3***	**3.2**	3.5*	-3.0
**Dyrk1b**	**20.0****	**2.4***	4.8	**5.8**	**3.5***	3.7*	-2.7
**Cacna1c**	**18.8**	**2.2**	4.6	**4.6**	**3.2**	3.9	-2.8
**Frs3**	**18.3****	**2.3***	2.9	**6.7***	**3.3***	3.7**	-2.5
**Zscan10**	**17.8****	**2.7***	4.0	**5.4**	**3.9***	3.3*	-2.9
**Myod1**	**14.3****	**2.9***	5.0	**8.8***	**3.2***	2.6*	-2.5
**Olfr1410**	**14.5****	**2.9****	3.6	**3.4**	**3.9****	3.4**	-2.5
**P2rx2**	**14.0****	**2.2***	3.8	**3.8***	**3.6****	2.8*	-2.3
**Shc2**	**13.2****	**2.3**	3.6	**4.2***	**3.2****	3.5**	-2.5
**Ybx2**	**12.8**	**2.5***	3.6	**5.9***	**3.4***	3.2*	-2.7
**Stag2**	**12.6**	**2.7**	3.8	**4.3**	**3.5***	3.3*	-2.8
**Sox15**	**12.3****	**2.2***	3.5	**6.3***	**3.5****	3.1**	-2.5
**Prp2**	**12.1****	**2.0**	3.8	**3.6***	**3.4****	2.6*	-3.1
**Syt16**	**12.0****	**1.8***	3.1	**3.9**	**3.1****	2.2	-1.7
**Dennd3**	**11.7****	**2.6***	3.9	**4.2**	**3.4***	3.3**	-2.5
**Tmc1**	**11.7****	**2.4****	3.4	**4.7***	**3.8****	2.9**	-2.3
**Dpf1**	**11.6****	**2.6**	3.8	**3.9**	**3.8****	2.7*	-2.5
**Spnb4**	**11.1****	**2.3***	3.4	**4.8**	**3.9****	3.0*	-2.3
**Wdr40c**	**11.0****	**2.3***	3.9	**2.5**	**3.5****	3.2**	-2.3
**Bcorl1**	**11.0**	**2.4***	3.3	**2.5**	**3.2***	2.8*	-2.4
**Col2a1**	**9.8****	**2.0***	3.1	**4.4***	**3.2****	2.2	-2.1
**Prh1**	**9.7****	**1.8**	3.7	**2.9**	**3.0***	2.0	-1.9
**Gprc5a**	**9.6****	**1.8**	3.4	**2.8**	**3.0****	2.8*	-1.9
**Cyp4a10**	**9.6****	**2.0***	2.7	**4.3**	**3.2****	2.4*	-2.2
**Rdh9**	**9.3****	**2.0**	2.9	**3.6***	**3.5****	2.2	-2.1
**Nlrp2**	**9.2****	**2.0***	3.1	**3.7**	**3.4****	2.4*	-2.0
**H1fx**	**9.1****	**4.2****	2.0	**nc**	**3.3***	1.3	-2.2
**Trp53**	**8.8****	**2.1***	3.2	**3.8**	**3.4****	2.8**	-2.0
**Pip5k1c**	**8.8****	**1.9***	3.4	**4.4***	**3.3****	2.6**	-1.9
**Pscd4**	**8.5****	**2.0**	3.1	**3.3**	**3.4****	2.0	-2.1
**Atp7b**	**8.2****	**2.1****	3.3	**3.2**	**3.6****	2.6**	-1.9
**Sall4**	**8.2****	**1.8***	3.0	**2.8**	**3.6****	2.1*	-1.9
**Chd4**	**8.1****	**1.8**	3.0	**2.6**	**3.0****	2.5*	-1.8
**Nestin**	**7.9****	**2.1****	3.0	**4.3****	**3.2****	2.3**	-2.0
**Trp73**	**7.6****	**1.9***	3.0	**3.6**	**3.2****	2.4**	-1.9
**Hoxc4**	**7.5****	**2.0***	2.8	**3.7**	**3.7****	2.4*	-2.0
**Rhot2**	**7.3****	**2.5***	2.7	**4.4**	**3.0***	3.3**	-2.5
**Fgl1**	**6.8****	**1.8***	3.2	**1.7**	**3.1****	2.4*	-2.1
**Vegfb**	**6.6****	**1.7**	2.9	**2.8**	**1.9**	2.1	-1.6
**Slc14a2**	**6.3**	**2.0***	2.1	**1.8**	**3.5****	1.7	-1.7
**Pex11c**	**6.3****	**2.4***	2.4	**2.8**	**3.8***	1.4	-1.8
**Pak6**	**6.0****	**1.9****	2.3	**2.7***	**3.1****	1.9**	-1.8
**Esrrg**	**5.8****	**1.9****	2.2	**3.5****	**3.2****	2.0**	-1.8
**Plxna1**	**5.7****	**1.8***	2.4	**2.6***	**3.0****	2.2*	-1.8
**Rdh12**	**5.7****	**1.8***	2.5	**2.9**	**3.0****	1.9	-1.9
**Sprr2b**	**5.6****	**1.9***	2.5	**2.3**	**2.9****	2.0*	-1.8
**Il17rd**	**5.5****	**1.7**	4.5*	**2.8***	**3.0****	2.1*	-1.7
**Defcr3**	**5.5****	**1.6**	2.7	**2.5**	**3.0****	2.2*	-2.0
**LactoP (Lpo)**	**5.4****	**1.8****	2.1	**2.4**	**3.0***	1.8**	-1.7
**Mdk**	**5.3****	**2.1****	2.2*	**3.5**	**3.5****	2.0*	-2.1

**Slc22a12**	**4.8****	**1.6***	2.5	**1.9**	2.8**	2.0**	-1.6
52							
**Adh/NA low**							
**Ecel1**	**13.0****	1.3	5.6	**3.4**	**2.2**	2.3	-1.7
**Glb1l**	8.7**	1.4	3.7	**2.8***	**3.0***	3.1**	nc
**Hoxb9**	**8.3****	1.5	3.6	**3.3#**	**3.0****	3.2**	nc
**Pcp2**	**7.9****	**1.7***	3.0	**2.1**	**2.9****	2.0	-1.5
**Cpeb2**	**7.6****	1.5	3.5*	**2.0**	**2.8***	3.0**	nc
**Rgs3**	7.0**	**1.4**	3.0*	**2.8***	**2.3****	2.0	-1.5
**Mid1**	**5.6****	**1.4**	3.0	**1.7**	**3.4****	1.7	nc
**Vash2**	**5.2****	**1.4**	3.4*	**2.4**	**2.4**	2.7**	nc
**Runx1**	**4.9****	**1.6***	2.5	**2.1#**	**2.2****	1.8*	-1.4
**Crtc3**	**4.7****	**1.6***	2.5	**1.9**	**2.4****	1.8*	-1.5
**Guca1b**	**4.6****	**1.5**	2.4	**2.2**	**3.1****	1.8*	nc
**Hoxb6**	**3.7****	**1.5**	2.3*	**1.9**	**2.9***	1.7*	nc
**Rgs1**	**3.3****	**1.3**	1.9**	**3.1****	**2.0****	3.4**	nc
**Il27**	**3.0****	**1.4**	1.9	**1.7**	**2.3****	1.5	-1.3
13							

Table S2B. DKO(-) sub-cluster.

Change vs WT control	**1b1$^{-/-}$ WT**	1a1$^{-/-}$ WT	1a1$^{-/-}$ BP 1A1$^{-/-}$	**DKO WT**	DKO BP WT	**WT TCDD WT**	WT BP WT
**Stimulation**							
Creb3l3	**13.5****	3.4**	2.0	**nc**	2.0	**3.8****	1.5
Duox2	**12.8****	3.3**	1.7	**nc**	1.8	**3.7****	1.6
Chrm4	**12.8****	2.4**	1.9	**nc**	1.8	**3.3****	1.7
Ppp1r14c	**12.7****	3.4*	2.0*	**nc**	1.8	**4.3****	1.6
Ptprz1-v	**12.2****	2.8*	1.45	**nc**	**2.2**	**3.2****	1.6
Syt6	**12.0****	2.6**	1.7	**nc**	**2.1****	**3.1****	1.5
**Slc25a35**	**11.0****	2.7**	1.5	**nc**	2.4	**2.9****	1.4
Adamtsl3	**11.4****	2.4**	1.7	**nc**	2.3	**3.0****	1.4
Kif12	**11.4****	2.7**	1.5	**nc**	2.2	**3.05****	**1.6**
Adamts4	**10.5****	2.6**	1.5	**nc**	2.4	**2.75****	1.5
Lgr4	**11.1****	3.0**	1.5	**nc**	2.1	**2.7****	1.3
Sin3b-v	**10.9****	2.5**	1.4	**nc**	**2.4***	**3.0****	1.5
Terc	**10.6****	2.8**	1.5	**nc**	2.1	**3.5****	1.5
Erv3	**10.4****	2.3**	1.5	**nc**	2.1	**2.8****	1.5
NeuroD2	**10.3****	2.8**	ns	**nc**	2.4	**2.8****	1.4
Cpb2	**10.3****	2.5**	1.6	**nc**	1.95	**2.7****	**1.3**
Acot6	**9.9****	2.7*	nc	**nc**	**1.75**	**2.3***	1.4
Evx2	**9.8****	1.9*	1.9*	**nc**	2.0	**2.5****	1.5
Ccl19	**9.5****	2.0**	1.7	**nc**	1.9	**2.9****	1.5
Trim68	**9.3****	2.0**	1.4	**nc**	2.0	**3.3****	1.4
Rad51l3	**7.4****	2.4	nc	**nc**	**1.95**	**3.0****	1.45

Table S2C. *Cyp1b1*$^{-/-}$ DKO(+) expression relative to common standard (WT NA).

Gene	WT Cy5	WT-AD NA	1a1$^{-/-}$ NA	1b1$^{-/-}$ NA	1b1$^{-/-}$ NA	1a1$^{-/-}$ BP NA	1a1$^{-/-}$ BP NA	DKO NA	DKO BP NA
**DKO(+) Cluster**	>3500 <1500			T1	T2	T1	T2		T1
Kcnh4	1021	-3.7	1.6	10.3	9.2	14.9	9.4	4.7	5.0
Polr2a	3198	-3.5	1.8	10.0	9.4	15.3	10.1	3.2	2.9
Ugt2b34	2177	-3.0	1.7	10.7	8.8	14.0	11.8	2.7	3.2
Cacna1c	742	-2.8	1.7	8.6	8.1	13.5	8.5	3.5	3.9
Dyrk1b	4838	-2.7	1.3	8.9	8.5	14.8	8.8	2.5	2.3
Frs3	2773	-2.5	1.4	8.3	8.0	15.5	9.0	3.0	3.0
Zscan10	2277	-2.9	1.4	7.4	6.2	10.5	7.2	2.0	1.8
Myod1	1476	-2.5	1.5	5.6	5.6	13.8	4.8	1.9	1.7
Olfr1410	5065	-2.5	1.4	7.4	5.6	10.6	6.8	1.5	1.5
P2rx2	1595	-2.3	1.2	7.1	5.9	9.3	6.7	1.8	1.7
**Shc2**	**14064**	-2.5	1.3	6.1	5.7	10.6	4.7	1.9	2.0
Ybx2	2108	-2.7	1.3	5.5	4.7	9.7	5.0	2.4	2.2
Stag2	7109	-2.8	1.4	5.3	5.2	10.7	6.2	1.8	1.9
Sox15	1326	-2.5	1.4	5.8	5.1	7.8	5.4	2.7	2.6
Prp2	2749	-3.1	1.2	6.4	6.1	10.2	5.7	1.9	1.9
Syt16	554	-1.7	1.3	4.7	4.2	7.0	5.3	2.4	1.9
Dennd3	4898	-2.5	1.4	6.2	4.4	11.7	7.3	1.9	2.7
Tmc1	3624	-2.3	1.3	5.7	5.2	9.0	5.3	2.2	2.0
Dpf1	3597	-2.5	1.3	5.7	4.6	9.0	7.5	1.7	1.6
**Spnb4**	**10196**	-2.3	1.3	5.6	4.9	9.1	5.2	2.3	2.1
**Wdr40c**	**16798**	-2.3	1.3	5.3	4.8	10.1	6.1	1.2	1.5
Bcorl1	7542	-2.4	1.2	5.4	4.6	9.1	4.6	1.1	1.4
Col2a1	1367	-2.1	1.1	5.7	4.6	6.1	5.2	2.3	1.8
Prh1	2428	-1.9	1.2	5.7	5.4	9.1	5.5	1.7	1.8
Gprc5a	4318	-1.9	1.3	5.9	5.1	8.7	5.0	1.6	1.8
Cyp4a10	1188	-2.2	1.2	5.0	4.7	6.5	3.3	2.2	2.2
Nlrp2	5973	-2.0	1.2	5.2	4.6	7.0	4.8	2.0	1.7
H1fx	99100	-2.4	2.2	4.8	4.1	6.4	4.5	-1.7	nc
**Trp53**	**10691**	-2.0	1.3	5.0	4.5	7.4	5.2	2.0	2.0
Pip5k1c	3921	-1.9	1.2	4.8	4.8	9.3	5.0	2.4	2.2
**Pscd4**	**26267**	-2.1	1.2	5.1	4.0	6.8	4.9	1.8	1.6
Atp7b	5391	-1.9	1.2	5.1	4.0	8.2	5.4	1.8	1.8
Sall4	4236	-1.9	1.1	5.0	4.3	6.3	4.2	1.6	1.4
**Chd4**	**29517**	-1.8	1.2	4.9	4.6	7.8	3.9	1.5	1.5
Nestin	1101	-2.0	1.2	4.3	3.4	7.1	4.4	2.2	2.1
Trp73	1250	-1.9	1.2	4.6	3.8	7.0	4.5	2.0	1.8
Hoxc4	3624	-2.0	1.2	4.2	3.8	6.5	4.0	2.0	1.8
Rhot2	4931	-2.5	1.4	3.2	3.3	7.5	3.1	2.0	2.7
**Fgl1**	**10196**	-2.1	1.2	3.8	3.0	6.7	3.0	-1.2	-1.1
Slc14a2	2768	-1.7	1.2	4.7	3.8	4.7	3.7	1.2	1.5
Pex11c	7056	-1.8	1.1	3.1	2.7	4.5	3.4	1.3	1.3
Pak6	1175	-1.8	1.1	3.5	3.2	4.7	2.9	1.5	1.2
Esrrg	2095	-1.8	1.2	3.3	3.2	3.9	3.0	2.0	1.5
Plxna1	3387	-1.8	1.1	3.7	3.2	4.9	3.1	1.6	1.3
Il17rd	2364	-1.7	1.1	3.5	3.1	5.4	3.4	1.6	1.5
Vegfb	7308	-1.6	1.3	3.9	4.7	7.0	4.1	1.4	1.6
**Rdh12**	**11149**	-1.9	1.1	3.3	3.0	5.5	2.7	1.6	1.4
**Sprr2b**	5581	-1.8	1.2	3.6	3.1	5.3	3.4	1.4	1.3
**Defcr3**	**33724**	-2.0	1.1	3.6	3.2	5.2	3.7	1.6	1.3
Lpo	3577	-1.7	1.1	3.4	3.1	4.6	3.3	1.4	1.3
Mdk	2150	-2.1	1.1	2.8	2.7	5.1	2.5	1.8	1.6
Runx1-v	1426	-1.4	1.2	3.9	3.4	4.9	4.0	1.6	1.0
Crtc3	756	-1.5	1.1	4.0	2.8	4.0	4.8	1.3	1.1

**Slc22a12**	**10954**	-1.6	1.1	**3.4**	**2.7**	**4.6**	**3.4**	1.2	1.1
**DKO(+)[NA]**									
Ecel1	**958**	-1.7	1.2	**10.8**	**7.9**	**12.8**	**12.2**	2.5	2.4
Glb1l	7597	1.0	1.0	**8.8**	**6.7**	**11.9**	**5.8**	2.5	2.2
Hoxb9	4756	1.0	1.2	**8.9**	**6.5**	**13.8**	**5.7**	3.0	2.4
Cpeb2	2029	1.0	1.0	**8.2**	**6.9**	**13.0**	**4.4**	1.9	2.0
Vash2	**1232**	-1.0	1.0	**5.6**	**5.0**	**11.3**	**5.0**	2.5	1.8
Pcp2	2738	-1.5	1.2	**6.2**	**5.1**	**7.8**	**5.0**	1.4	1.7
Mid1	7920	-1.0	1.2	**5.5**	**3.7**	**6.2**	**5.3**	1.4	1.2
**Rgs3**	**12476**	-1.5	-1.1	**5.0**	**4.6**	**5.9**	**3.7**	1.9	1.4
Guca1b	3056	1.0	1.3	**4.0**	**3.4**	**5.7**	**3.8**	1.8	1.6
Hoxb6	3077	1.0	1.3	**3.5**	**3.1**	**5.2**	**3.6**	1.7	1.5
Rgs1	2094	1.0	-1.1	2.8	2.8	**2.4**	**2.4**	2.6	2.4
Il27	962	1.3	1.1	2.6	2.3	**3.3**	**2.4**	1.4	1.5

Table S2D. DKO(-) expression relative to common standard (WT NA).

**DKO(-)**	Cy5 NA **>1500**	WT NA	1a1$^{-/-}$ NA	**1b1$^{-/-}$ NA T1**	**1b1$^{-/-}$ NA T2**	**1a1$^{-/-}$ BP NA T1**	**1a1$^{-/-}$ BP NA T2**	DKO NA	DKO BP NA T1/T2
Creb3l3	**2112**	**-1.4**	2.9	**12.5**	**8.2**	**5.8**	**4.5**	nc	1.5/2.6
Duox2	**1526**	**-1.4**	2.8	**11.0**	**8.1**	**5.8**	**4.5**	nc	1.7/2.7
Chrm4	**1139**	**-1.4**	1.7	**11.5**	**7.9**	**5.6**	**4.2**	nc	1.6/2.3
Ppp1r14c	**3400**	**-1.6**	2.2	**10.1**	**7.5**	**5.4**	**4.4**	nc	1.5/2.3
Slc25a35	**990**	**-1.1**	2.6	**11.4**	**7.9**	**3.9**	**3.8**	nc	1.5/2.1
Adamtsl3	**952**	**-1.6**	1.9	**12.2**	**8.0**	**4.5**	**3.8**	nc	1.6/2.4
Kif12	**832**	**-1.2**	2.6	**11.8**	**8.2**	**4.9**	**3.7**	nc	1.8/2.5
Adamts4	**878**	**-1.2**	2.2	**11.0**	**7.7**	**4.4**	**3.6**	nc	1.5/2.2
Lgr4	**845**	**-1.2**	3.4	**11.5**	**8.5**	**4.4**	**3.8**	nc	1.6/2.3
Terc	**1139**	**-1.4**	2.2	**8.9**	**7.0**	**4.7**	**3.6**	nc	1.4/2.2
Erv3	**473**	**-1.2**	1.6	**11.2**	**6.6**	**4.6**	**3.6**	nc	1.6/2.3
NeuroD2	**577**	**-1.2**	2.8	**10.9**	**7.1**	**3.5**	**3.6**	nc	1.6/2.3
Cpb2	**966**	**-1.2**	2.3	**10.5**	**7.5**	**4.4**	**3.9**	nc	1.3/2.1
Evx2	**862**	**-1.1**	2.3	**10.9**	**7.5**	**4.8**	**3.4**	nc	1.8/2.6
Ccl19	**1065**	**-1.2**	1.8	**9.1**	**6.8**	**4.8**	**3.7**	nc	1.6/2.4
Trim68	**1909**	**-1.3**	2.8	**9.3**	**6.1**	**4.1**	**3.7**	nc	1.6/2.4
Rad51l3	**1342**	**-1.2**	2.8	**7.7**	**4.9**	**3.8**	**2.8**	nc	1.4/1.8
Acot6	**563**	**1.0**	2.8	**11.9**	**8.4**	**4.4**	**3.2**	nc	1.5/2.2
Sin3b-v	**663**	**-1.2**	2.1	**11.2**	**7.4**	**4.0**	**3.6**	nc	1.7/2.4
Ptprz1-v	**854**	**-1.3**	2.8	**12.0**	**8.5**	**4.5**	**4.3**	nc	1.5/2.1
Syt6	**917**	**-1.2**	2.0	**11.9**	**8.4**	**4.9**	**4.0**	nc	1.6/2.0

Table S3. Cyp1b1-AhR: OLFACTORY RECEPTORS (OLFR).

Table S3A. LIMMA Ratios to WT:*Cyp1b1*$^{-/-}$ versus *Cyp1a1*$^{-/-}$ BP and TCDD.

		1b1	1a1$^{-/-}$ BP	DKO BP	TCDD
T/WT	Cy3>1000	WT	WT[a]	WT	WT
1026	811	7.0	2.3	3.2**	3.2**
1030	366	7.7	2.9*	2.5	2.5**
1048	3213	4.6	3.4	2.1	3.1**
1131	690	3.0	2.5	1.8	2.0
1134	299	3.6	2.8	1.7	2.9
1143	284	4.6	3.4	2.4	2.8
1153	2880	4.4	2.8	nc	1.8
1198	277	2.8	1.8	nc	1.5
123	153	8.5	3.7	2.8	2.4
127	491	10.0	2.8	1.8	2.4*
1293	892	3.4	3.6	nc	2.7
1339	323	4.0	3.0	2.7	3.1**
1359	290	11.5	3.8	2.5	2.1
139	133	9.6	6.5	3.0	2.5
1394	2691	4.8	4.3	3.3	3.2**
1410	5065	14.6	10.3	4.2*	3.9**
1444	605	4.3	3.7	2.1	2.1
1459	603	13.9	6.8	6.6*	3.4
149	272	4.1	3.0	1.5	2.4*
1495	4274	1.5	1.3	nc	nc
1507	1998	6.1	3.2	1.8	2.1
166	541	6.2	4.1	2.8**	2.8
187	283	2.9	2.9	nc	2.1
234	2619	5.8	4.9	3.0**	3.0**
414	706	6.9	4.6	3.5*	2.7
430	2255	2.4	1.6*	nc	1.9*
469	450	1.2	nc	nc	nc
508	904	8.8	5.9	2.6	2.8*
544	635	3.9	3.2	1.9	2.3*
549	532	4.1	3.6	2.2	2.5**
570	960	8.3	5.9	3.4	3.1**
571	11810	3.1	3.0	1.4	2.6**
59	364	4.1	3.0	2.5	2.1**
611	717	12.4	8.3	6.5**	3.3
62	1195	4.3	3.5	2.1	3.0**
656	337	5.2	3.6	2.8	2.8*
661	197	3.5	2.0	nc	1.7
684	386	10.8	6.8	5.4*	2.7
741	616	3.8	1.7	nc	1.6
744	2916	3.0	2.9	1.9	2.9**
780	808	16.1	6.9	3.3	4.5**
821	2320	15.5	10.2	6.5**	3.3**
933	804	4.0	3.1	1.6	2.4**
974	346	8.4	5.4	4.2**	3.7**
>5<8					
>8					
>3<5					

Table S3B. OLFR Division between DKO(+) and DKO(-): Individual Mouse Treatments Cy3, Ratio to NA Cy5.

		WT-1	TCDD	BP	1b1$^{-/-}$	1a1$^{-/-}$	1a1$^{-/-}$BP	DKO	DKO-BP
Olfr	Cy5 (NA)	Adh/NA Cy3/Cy5	WT=NA	WT-NA	WT-NA INDV	WT-NA	WT-NA[a] INDV	WT-NA	WT-NA INDV
DKO(+)									
Polr2a	2896	-5.1	1.5	1.8	9.8/8.4	nc	15.7/9.5	2.7	2.5/3.1
611	717	-3.5	1.7	1.5	6.6/5.2	nc	8.3/8.1	3.4	3.1/3.9
821	2320	-3.6	1.6	1.6	7.6/6.5	nc	10.4/8.0	2.4	2.7/3.3
1459	603	-3.6	1.5	1.3	6.9/5.5	nc	8.6/8.0	2.2	2.8/3.0
1410	5065	-3.5	1.8	1.6	7.4/5.6	nc	10.6/6.8	1.5	1.5/2.3
508	904	-2.9	1.7	1.6	5.8/4.6	nc	7.6/5.0	1.5	1.5/1.9
1026	811	-2.5	1.8	1.3	4.4/3.4	nc	6.3/5.0	1.5	1.5/2.0
570	960	-2.3	1.7	nc	4.9/4.2	nc	7.6/4.5	1.8	1.6/2.0
684	386	-2.2	1.5	1.4	6.6/4.9	nc	7.6/6.2	2.5	2.7/3.0
414	706	-2.2	1.6	1.4	4.7/3.6	nc	5.8/2.9	2.3	1.9/2.2
974	346	-2.2	1.7	nc	4.3/3.6	nc	4.0/4.4	1.9	1.7/2.2
1030	366	-1.7	1.6	1.4	5.5/4.3	nc	5.8/5.9	1.7	1.6/2.0
234	2619	-2.0	1.9	1.3	3.7/3.4	nc	6.2/4.1	2.1	1.5/2.0
544	635	-1.9	1.5	nc	2.7/2.5	nc	3.7/2.7	1.3	1.3/1.4
656	337	-1.8	1.7	nc	3.4/3.0	nc	3.8/3.0	1.7	1.5/1.8
549	532	-1.6	1.7	nc	2.9/2.5	nc	4.5/2.8	1.5	1.2/1.9
166	616	-1.5	1.5	nc	3.7/2.9	nc	3.4/2.7	1.7	1.4/1.7
1048	3213	-1.6	2.1	nc	3.2/2.9	nc	3.5/2.5	1.4	1.2/1.6
59	364	-1.4	1.5	1.6	3.0/2.9	nc	4.3/2.5	1.8	1.3/1.7
62	1195	-1.7	1.9	nc	3.1/2.3	nc	3.4/2.8	1.3	1.1/1.4
744	2916	-1.3	2.4	1.5	2.8/2.3	nc	3.6/3.7	1.3	1.3/2.0
1339	323	-1.9	1.7	nc	2.3/2.1	nc	2.4/2.4	1.5	1.2/1.7
DKO(-)									
Creb3l3	2112	-2.2	2.9	1.6	12.5/8.2	2.6	5.8/4.6	nc	1.5/2.6
780	808	-2.3	2.1	1.9	10.3/5.6	1.4	6.6/3.3	nc	nc/2.0
1359	290	-1.2	2.1	1.7	10.3/6.4	1.4	4.2/3.4	nc	1.5/2.0
127	491	-1.3	2.1	2.2	10.5/6.9	1.3	5.0/3.4	nc	1.3/1.8
1293	892	-1.7	2.5	1.5	6.0/14.1	1.5	6.9/2.3	nc	nc/1.7
1507	1998	-1.5	2.0	2.0	5.9/5.2	1.4	6.6/2.2	nc	1.5/1.9
1153	2889	1.2	2.0	2.6	4.8/3.9	1.4	2.0/3.6	nc	nc/1.5
741	616	1.2	2.1	2.4	4.2/3.4	1.3	3.6/3.7	nc	nc/1.5
Basal Low Induction									
571	11810	-1.5	2.0	nc	2.7/2.2	nc	1.9/1.6	nc	nc/1.4
933	804	-1.3	1.8	1.3	3.2/3.0	nc	3.7/3.3	nc	nc/1.4
1444	605	-1.6	1.3	nc	2.7/2.6	nc	3.7/2.9	nc	nc/1.4
1394	2691	-2.3	1.5	nc	2.3/2.1	nc	4.7/1.6	nc	1.5/1.6
430	2255	1.2	2.0	1.8	2.7/2.2	nc	1.8/1.6	nc	nc/1.4
1495	4274	1.2	1.4	2.1	1.6/1.6	nc	1.7/2.3	nc	nc/1.5
Standards									
Gapdh	34073	1.0	nc	nc	nc/nc	nc	nc/nc	nc	nc/nc
ActB	48904	-1.1	nc	nc	nc/nc	nc	nc/nc	nc	nc/nc

Table S4. RNA POLYMERASE 2 (POLR2). 12 Treatment Matrix for Individual Mice. Comparison of 12 POLRA Subunits.

Subunit	A	B	C	D	E	F	G	H	I	J	K	L
Ref	NA	NA	NA	NA	NA	NA	NA	NA	NA	NA	NA	NA
WT treat												
WT	-5.1	nc	nc	nc	nc	nc	nc	nc	nc	-1.5	nc	nc
WT	-1.4	nc	nc	nc	nc	nc	nc	nc	nc	nc	nc	nc
WT	-2.8	1.4	nc	nc	-1.3	nc	-1.3	-1.3	-1.6	-1.8	nc	-1.4
T12	nc	nc	nc	nc	nc	-1.5	nc	-1.4	nc	-1.9	nc	-1.3
T12	2.9	nc	nc	nc	nc	-1.7	nc	-1.6	nc	-1.6	nc	-1.4
T12	nc	-1.4	nc	1.3	nc	nc	nc	nc	nc	nc	nc	nc
BP6	1.4	nc	nc	-1.3	-1.3	-1.6	-1.3	-1.7	nc	-1.5	nc	-1.5
BP6	1.3	nc	nc	nc	nc	-1.4	-1.4	-1.8	-1.5	-1.5	-1.3	-1.5
BP12	nc	1.3	nc	nc	nc	-1.3	nc	-1.4	nc	-1.3	nc	nc
BP12	1.4	1.5	nc	nc	nc	-1.4	-1.3	-1.4	nc	-1.4	nc	nc
BP12	3.0	nc	-1.4	-1.5	-1.6	-1.4	nc	-1.3	nc	nc	nc	nc
BP24	nc	nc	nc	-1.8	nc	-1.4	-1.6	-1.4	nc	-1.3	nc	-1.4
BP24	nc	1.5	nc	-1.3	nc	nc	-1.3	-1.6	nc	-1.4	nc	nc
BP24	2.0	nc	nc	-1.4	nc	nc	-1.3	-1.6	nc	nc	1.3	-1.3
DB12	-2.0	nc	nc	nc	nc	nc	nc	-1.4	nc	nc	nc	nc
DB12	-1.6	-1.3	nc	nc	nc	nc	nc	-1.4	nc	nc	nc	nc
DB12	-2.8	1.3	nc	nc	nc	-1.3	nc	-1.3	-1.3	-1.6	nc	-1.4
Cyp1												
1BK	9.8	-1.3	-1.4	-1.4	-1.4	nc	1.4	nc	1.3	nc	1.4	nc
1BK	8.4	nc	nc	nc	-1.3	nc	1.5	nc	1.4	nc	1.5	nc
1BKDB	3.1	nc	nc	-1.7	-1.7	nc	nc	-1.3	nc	nc	nc	-1.4
1BKDB	2.7	1.0	-1.3	-1.5	-1.6	nc	nc	-1.3	nc	nc	nc	-1.3
1AK	1.7	nc	-1.3	-1.4	-1.3	-1.5	nc	-2.0	nc	-1.6	nc	-1.6
1AK	nc	-1.4	-1.4	-1.7	-1.5	-1.5	nc	-2.3	nc	-1.6	nc	-1.7
1AK	-1.3	-1.5	-1.4	-1.5	-1.6	-1.9	nc	-2.4	-1.4	-2.2	nc	-1.8
1AKBP	nc	-1.4	-1.4	-1.6	-1.5	-1.8	nc	-2.7	-1.4	-1.9	1.3	-1.8
1AKBP	15.7	-1.3	nc	-1.5	nc	-1.7	nc	-2.2	nc	nc	nc	-1.3
1AKBP	9.5	nc	nc	-1.3	nc	nc	1.3	-1.6	1.4	nc	1.3	-1.2
DKO	2.7	nc	nc	nc	-1.3	nc	nc	nc	nc	nc	nc	nc
DKOBP	2.5	nc	nc	nc	-1.3	-1.3	-1.3	-1.6	nc	nc	-1.3	-1.4
DKOBP	3.1	-1.3	nc	nc	nc	nc	nc	-1.6	nc	nc	nc	-1.3

Table S5. 12 Treatment Matrix MARKER genes for different clusters.

gene	Ahrr	Spint1	Ptgs2	Il1b	Tnf	Hp70	Pol2a	Nes	Olr1410	Creb3l3	Igh-2
cluster	Can	Can	BP1A1	BP1A1	BP1A1	1a1$^{-/-}$	DKO+	DKO+	DKO+	DKO-	1b1sp
WT	4.5	3.1	2.8	1.6	1.3	nc	-5.4	-2.4	-3.5	-2.2	nc
WT	nc	nc	nc	nc	1.3	nc	-1.5	-1.3	-1.2	nc	nc
WT	2.8	2.9	3.6	1.6	1.4	nc	-3.1	-2.2	-2.7	nc	nc
T12	7.6	9.6	1.5	1.8	2.2	9.6	nc	1.7	1.8	3.3	-1.3
T12	10.2	10.9	3.6	2.7	3.7	9.5	2.9	2.1	2.3	2.2	1.8
T12	10.5	10.4	nc	nc	2.4	6.7	-1.3	1.4	1.3	3.3	1.4
BP6	21.0	11.3	14.2	8.0	8.2	1.4	1.4	nc	nc	nc	1.6
BP6	20.3	10.4	19.6	5.1	7.3	nc	1.3	nc	nc	nc	1.2
BP12	11.5	10.8	6.9	4.0	4.4	1.4	nc	nc	nc	-1.4	2.1
BP12	3.7	6.7	5.6	2.3	3.5	nc	1.4	nc	1.4	-2.2	1.6
BP12	7.3	8.2	6.8	5.2	5.0	1.7	3.0	1.7	2.5	4.2	1.9
BP24	14.4	12.2	2.1	-3.0	1.3	2.6	nc	nc	nc	-1.4	2.9
BP24	20.8	16.7	1.5	-3.0	nc	nc	nc	nc	nc	-1.9	2.3
BP24	20.0	10.8	1.3	-3.5	-1.5	1.4	1.9	nc	nc	-1.3	1.3
DB12	11.1	9.6	1.9	1.4	nc	nc	-1.9	nc	-1.5	1.4	1.5
DB12	11.6	9.4	1.9	1.4	nc	nc	-1.5	nc	nc	1.3	1.7
DB12	8.3	6.5	4.3	2.2	1.4	nc	-2.7	-1.5	-2.1	1.5	1.2
1BK	-3.4	-2.4	1.9	3.1	2.1	1.9	10.0	4.3	7.4	12.5	8.6
1BK	nc	-2.8	nc	nc	1.5	2.9	9.4	3.4	5.6	8.2	4.5
1BKDB	11.4	5.9	15.3	4.9	3.1	5.7	3.2	1.3	1.7	2.1	4.9
1BKDB	2.2	3.4	9.9	4.7	3.5	2.9	2.4	nc	1.3	1.6	5.2
1AK	nc	nc	3.7	2.5	3.9	10.6	1.8	nc	1.4	1.6	1.8
1AK	nc	nc	nc	nc	3.4	3.8	nc	nc	nc	3.4	3.1
1AK	nc	nc	nc	nc	3.8	3.5	nc	nc	nc	2.9	3.1
1AKBP	7.5	10.8	2.5	nc	2.7	3.9	nc	nc	1.3	2.4	2.8
1AKBP	9.3	10.0	2.9	3.5	2.6	10.0	15.3	7.1	10.6	5.8	1.9
1AKBP	22.4	14.5	4.4	4.2	2.8	6.4	10.1	4.4	6.8	4.6	1.2
DKO	1.8	-1.8	nc	1.4	3.1	2.6	3.2	2.2	1.5	1.0	3.5
DKOBP	23.7	17.1	nc	2.0	4.4	3.4	2.9	2.1	1.5	1.5	3.6
DKOBP	20.8	13.6	nc	nc	2.4	2.2	3.3	2.3	2.3	2.6	4.6

Table S6. Cyp1b1-TCDD Suppressions.

Limma	Adh	1b1$^{-/-}$	1a1$^{-/-}$	DKO	WT-TCDD	WT-BP
Ratio	NA	WT	WT	WT	WT	WT
Gene						
A. (BP active)						
Bcl11a*	1.6	-5.7**	-2.8**	-2.5**	-3.2	-2.6**
Fcrla	1.8	-4.4**	-1.9*	-3.8**	-2.4**	-1.7**
Clec2d	1.7	-4.4**	-1.4	-3.4*	-3.3	-2.8**
Reln	1.3	-4.1**	-1.8*	-3.1**	-2.1	-2.1**
Ndg2	1.6	-3.8**	-1.9*	-2.1*	-1.7**	-1.6**
Rag1	1.4	-3.6**	-1.6	-4.1**	-2.0*	-2.8**
CrK	1.3	-2.9**	-2.3**	-2.4**	-2.4**	-2.0**
Wnt10a	2.3	-2.6**	-2.1	-2.8*	-1.8	-2.1**
Vpreb3	1.5	-2.4**	-1.5	-2.7**	-1.9**	-2.0**
Ms4a1	1.5	-2.4**	-2.3**	-3.8**	-1.7	-2.3**
Lcor	1.7	-3.7**	-2.4**	-1.6	-2.6	-1.8*
Ddah2	1.5	-2.6**	-1.5	-1.9**	-1.7**	-2.1**
Pyhin1	1.2	-2.4**	-1.8	-3.8**	-2.0	-1.7
B. (BP inactive)						
Rapgefl1	1.5	-4.5**	-2.1	-3.1**	-2.7**	nc
Cbfa2t3	1.5	-3.7**	-2.3**	-1.9**	-2.5**	nc
Klhl6	1.5	-3.6**	-1.3	-2.8(1.4)	-2.4**	nc
Mlxip	1.9	-3.5**	-1.9	-2.1	-1.8*	nc
Mylip	1.8	-3.2**	-1.6	-3.2*	-1.8*	nc
Mycbp2	1.5	-3.1**	-1.4	-2.4	-2.0*	nc
Ccng1	1.5	-2.4**	-1.5**	-2.2	-2.3**	nc
C. (TCDD/BP Inactive)						
Lgals3	1.3	-2.0	-1.5	-1.9(1.5)	nc	nc
Cd79b	1.4	-3.5**	-1.6	-2.6**	nc	nc
Osteoblasts						
Col1a1	2.2	-2.8**	nc[a]	-1.6	-4.9**	-3.5**
Col1a2	2.0	-1.8**	nc[a]	-2.1*	-5.5**	-1.5*
Bglap1	1.8	-1.8**	nc[a]	-1.6**	-3.2**	-2.6
Spp1	1.0	-2.2**	-3.0**	-2.6*	-2.9**	*-4.0***
Erythroblast						
Late Only						
Epb4.1 cluster						
Epb4.1	2.6	-3.3*	-1.8	nc	-1.9	1.4
Cat	2.2	-1.8	-1.8*	nc	-2.0*	1.8*
Cdc25b	2.3	-2.5**	-1.8	nc	-2.1*	nc
Chfr	2.3	-2.5*	-2.0*	nc	-2.6*	nc
Zfp36l1	2.1	-2.2	-1.7	nc	-1.7	1.4
Hif1a	2.0	-1.8	-2.1*	nc	-3.1*	1.4
Tcf12	2.1	-2.6	-2.5	nc	-2.3*	nc
Ogt	2.3	-2.8	-2.7*	nc	-3.0*	nc
General						
Hbb-b1	1.2	2.3**	1.9**	-1.6	2.8**	2.1*
Hba-a1	1.25	1.5	1.8**	-1.7	2.3**	2.7**
Hba-a2	1.1	1.7*	1.8**	-1.8	2.5**	2.0*
Fau	1.1	2.1**	2.1**	-1.5	1.9**	1.4*
Rps14	1.0	2.2**	2.1**	-1.5*	1.9**	1.7*
S100a9	1.15	3.6**	1.9**	-1.7	1.8**	2.2**
Chi3l3	1.1	2.9**	2.0**	-1.6*	1.5**	2.1**

Table S7. BMS2 expression as guide to BM-MSC.

Table S7A. BM DKO(+) versus BMS2.

Gene	1b1$^{-/-}$	N-Ad	BMS2	Gene	1b1$^{-/-}$	N-A	BMS2
**STIMULATION Fold Change**	**WT**	**Cy5**	Cy3 FC	**STIMULATION Fold Change**	**WT**	**Cy3**	
Limma	p<01**						
**Kcnh4**	**30.7****	**1021**	**19**	**Hoxc4**	**7.5****	**3694**	**197**
**Polr2a**	**28.0****	**2896**	**190**	**Rhot2**	**7.3****	**4931**	**2128 2**
**Ugt2b34**	**25.7****	**2177**	**4**	**Fgl1**	**6.8****	**10196**	**8547 1**
**Dyrk1b**	**20.0****	**4838**	**344**	**Vegfb**	**6.6****	**7808**	**7300 1**
**Cacna1c**	**18.8****	**742**	**380 2**	**Slc14a2**	**6.3**	**2768**	**515 5**
**Frs3**	**18.3****	**2773**	**460**	**Pex11c**	**6.3****	**7056**	**2474 3**
**Zscan10**	**17.8****	**2277**	**140**	**Pak6**	**6.0****	**1175**	**88**
**Myod1**	**14.3****	**1329**	**50**	**Esrrg**	**5.8****	**2095**	**103**
**Olfr1410**	**14.5****	**5065**	**264**	**Plxna1**	**5.7****	**3387**	**3124 1**
**P2rx2**	**14.0****	**1595**	**250**	**Rdh12**	**5.7****	**11149**	**1781**
**Shc2**	**13.2****	**14064**	**1177**	**Sprr2b**	**5.6****	**5581**	**1400 4**
**Ybx2**	**12.8**	**2108**	**760 3**	**Il17rd**	**5.5****	**2364**	**476 5**
**Stag2**	**12.6**	**7109**	**2689 2.5**	**Defcr3**	**5.5****	**14869**	**4596 3**
**Sox15**	**12.3****	**1326**	**154**	**Lpo**	**5.4****	**3577**	**165**
**Prp2**	**12.1****	**2749**	**245**	**Mdk**	**5.3****	**2150**	**590 4**
**Syt16**	**12.0****	**554**	**30**	**Slc22a12**	**4.8****	**10954**	**1953 5**
**Dennd3**	**11.7****	**4898**	**298**				
**Tmc1**	**11.7****	**3644**	**75**				
**Dpf1**	**11.6****	**3597**	**534**				
**Spnb4**	**11.1****	**10205**	**665**	**No ADH bias**			
**Wdr40c**	**11.0****	**16798**	**3120 5**	**Ecel1**	**13.0****	**958**	**149**
**Bcorl1**	**11.0**	**7542**	**2532 3**	**Glb1l**	8.7**	**7597**	**1153**
**Col2a1**	**9.8****	**1367**	**8363**	**Hoxb9**	**8.3****	**4756**	**23**
**Prh1**	**9.7****	**2428**	**551 4**	**Pcp2**	**7.9****	**2738**	**509 5**
**Gprc5a**	**9.6****	**4318**	**3287 1.3**	**Cpeb2**	**7.6****	**2029**	**143**
**Cyp4a10**	**9.6****	**1188**	**56**	**Rgs3**	7.0**	**12476**	**5665 2.5**
**Rdh9**	**9.3****	**818**	**180 5**	**Mid1**	**5.6****	**7920**	**473**
**Nlrp2**	**9.2****	**5973**	**930**	**Vash2**	**5.2****	**1232**	**688 2**
**Trp53**	**8.8****	**10691**	**1251**	**Runx1**	**4.9****	**1426**	**401 3**
**Pip5k1c**	**8.8****	**3921**	**945 4**	**Crtc3**	**4.7****	**756**	**279 3**
**Pscd4**	**8.5****	**26267**	**5552 5**	**Guca1b**	**4.6****	**3056**	**430**
**Atp7b**	**8.2****	**5391**	**460**	**Hoxb6**	**3.7****	**3027**	**148**
**Sall4**	**8.2****	**4236**	**532**	**Rgs1**	3.3**	**2094**	**3**
**Chd4**	**8.1****	**29517**	**3757**			**Lo BMS2**	**Hi BMS2**
**Nestin**	**7.9****	**1101**	**380 3**	**13**			
**Trp73**	**7.6****	**1250**	**105**				
		**Lo BMS2**	**Hi BMS2**	**Ratio 1-8**			

Table S7B. BMS2 expressed OLFR (3 cultures) versus adherent BM WT (3 cultures).

		BMS2<br><br>control		BM		BM<br>WT-Adh		1b1$^{-/-}$	1a1$^{-/-}$<br>BP
Olfr	1	2	3	Adh/NA	1	2	3	(2)	(2)
**1410**	**264**	**334**	**231**	**-2.6**	**5065**	**1436**	**1350**	**6.5**	**8.7**
**1153**	**1101**	**998**	**1137**	**1.0(-)**	**2889**	**1989**	**1671**	**4.3**	**2.8**
**234**	**709**	**793**	**961**	**-1.7**	**2619**	**1126**	**1697**	**3.5**	**5.2**
**656**	**158**	**207**	**246**	**-1.8**	**196**	**263**	**114**	**3.7**	**3.5**
**166**	**242**	**301**	**253**	**-1.2**	**567**	**378**	**398**	**3.3**	**3.1**
**571**	**2832**	**3016**	**3569**	**-1.3**	**11810**	**4656**	**7180**	**2.4**	**3.3**
**549**	**246**	**284**	**284**	**-1.6**	**532**	**327**	**405**	**2.7**	**3.6**
**1143**	**160**	**174**	**116**	**-2.5**	**135**	**143**	**55**	**2.4**	**2.7**
**430**	**856**	**785**	**843**	**1.0**	**2255**	**2136**	**1558**	**2.4**	**1.8**
**187**	**606**	**578**	**815**	**-1.7**	**283**	**300**	**158**	**1.8**	**2.9**
**Defcr3**	**5088**	**5360**	**3339**	**-1.7**	**14869**	**7874**	**9924**	**3.4**	**4.5**
Gapdh	49332	46304	71400		34073	12808	24221		

Shared with islet cells.

Table S8. OLFR genes expressed in BM cells.
Comparison to Pancreatic Islets and MIN cells (reference 25 Supplementary Data).

Olfr ID	Islets EXP	MIN EXP	BM-WT EXP	1b1$^{-/-}$ STIM	1a1BP STIM	Olfr ID	Islets EXP	MIN EXP	BM-WT EXP	1b1$^{-/-}$ STIM	1a1BP STIM
15	21	130				656	25	28	337	3.2	3.5
53	23	80				661	70	44	197	4.2	2.8
59	30	20	364	3.0	3.4	738	16	171			
103	33	123				745	129	1783			
123	38	106	153	5.3	3.3	780	50	45	808	8.0	5.0
128	128	220				821	44	456	2320	7.0	9.2
139	19	37	133	5.3	6.2	978	18	26			
149	34	127	272	3.4	3.6	1030	24	70	366	4.9	5.8
159	21	67				1043	11	32			
166	40	57	541	4.2	3.6	1131	31	61	690	2.2	2.4
187	36	438	283	1.8	2.9	1134	15	53	299	2.3	2.4
297	16	69				1143	25	262	284	2.4	2.7
373	11	1464				1170	14	46			
456	14	43				1198	61	33	277	3.4	2.6
469	14	62	450	1.4	1.4	1222	163	127			
513	13	78				1344	16	18			
521	91	263				1350	42	225			
522	18	58				1384	242	111			
524	16	165				1393	86	80			
544	63	5743	635	2.6	3.2	1410	87	20	5065	6.5	8.7
549	155	574	532	2.7	3.6	1417	43	33			
559	39	35				1441	77	38	379	2.4	2.7
609	33	159				1443	56	156			
611	351	851	717	5.9	8.2						

Shared with BMS2 cells.
Relative expression numbers taken from supplemental data_Pancreatic paper.

Table S9. Primers used for rt-PCR analyses of BM macrophage.

	F	R
PPARG	ATG GAG CCT AAG TTT GAG TTT G	CAG CAG GTT GTC TTG GAT GTC
CD36	GCGACATGATTAATGGCACAG	GATCCGAACACAGCGTAGATAG
TNFα	GTCTACTGAACTTCGGGGTGAT	ATGATCTGAGTGTGAGGGTCTG
IRF-4	GACCAGTCACACCCAGAAATCCC	GTTCCTGTCACCTGGCAACC
H2-AB1	GATCTTCCTCGGGCTTGG	ATTCGGAGCAGAGACATTCAG
Fn1	CCTGCACCTGATGGTGAA	GCCAGTGATTGTCTCTGTCT
iNOS	GCAAACATCACATTCAGATCCC	TCAGCCTCATGGTAAACACG
Klf4	ACTTGTGACTATGCAGGCTG	ACAGTGGTAAGGTTTCTCGC
Cyp1b1	TCCAGCTTTTTGCCTGTCAC	TGGCTGGGTCATGATTCACA
IL-1β	ACGGACCCCAAAAGATGAAG	TTCTCCACAGCCACAATGAG

References

1. Larsen, M.C., et al., *Cytochrome P4501B1 in bone marrow is co-expressed with key markers of mesenchymal stem cells. BMS2 cell line models PAH disruption of bone marrow niche development functions.* Toxicology and Applied Pharmacology, 2020. **401**.
2. Liu, X.Q. and C. Jefcoate, *2,3,7,8-Tetrachlorodibenzo-p-dioxin and epidermal growth factor cooperatively suppress peroxisome proliferator-activated receptor-gamma 1 stimulation and restore focal adhesion complexes during adipogenesis: Selective contributions of Src, Rho, and Erk distinguish these overlapping processes in C3H10T1/2 cells.* Molecular Pharmacology, 2006. **70**(6): p. 1902-1915.
3. Maguire, M., et al., *Cyp1b1 directs Srebp-mediated cholesterol and retinoid synthesis in perinatal liver; Association with retinoic acid activity during fetal development.* Plos One, 2020. **15**(2).
4. Falero-Perez, J., et al., *CYP1B1: A key regulator of redox homeostasis.* Trends in cell & molecular biology, 2018. **13**: p. 27-45.
5. Yuan, J.J., et al., *Quantitative Profiling of Oxylipins in Acute Experimental Intracerebral Hemorrhage.* Frontiers in Neuroscience, 2020. **14**.
6. Fader, K.A., et al., *2,3,7,8-Tetrachlorodibenzo-p-dioxin dose-dependently increases bone mass and decreases marrow adiposity in juvenile mice.* Toxicology and Applied Pharmacology, 2018. **348**: p. 85-98.
7. N'Jai, A.U., et al., *Bone marrow lymphoid and myeloid progenitor cells are suppressed in 7,12-dimethylbenz(a)anthracene (DMBA) treated mice.* Toxicology, 2010. **271**(1-2): p. 27-35.

8. Fu, Z.D., et al., *RNA-Seq Profiling of Intestinal Expression of Xenobiotic Processing Genes in Germ-Free Mice.* Drug Metab Dispos, 2017. **45**(12): p. 1225-1238.
9. Seok, S.H., et al., *Trace derivatives of kynurenine potently activate the aryl hydrocarbon receptor (AHR).* Journal of Biological Chemistry, 2018. **293**(6): p. 1994-2005.
10. Wilson, R.H. and C.A. Bradfield, *Rodent genetic models of Ah receptor signaling.* Drug Metab Rev, 2021. **53**(3): p. 350-374.
11. Heidel, S.M., et al., *Cytochrome P4501B1 mediates induction of bone marrow cytotoxicity and preleukemia cells in mice treated with 7,12-dimethylbenz[a]anthracene.* Cancer Res, 2000. **60**(13): p. 3454-60.
12. Bredemeyer, A.L., et al., *DNA double-strand breaks activate a multi-functional genetic program in developing lymphocytes.* Nature, 2008. **456**(7223): p. 819-U113.
13. Nebert, D.W., et al., *Oral benzo[a]pyrene: understanding pharmacokinetics, detoxication, and consequences--Cyp1 knockout mouse lines as a paradigm.* Mol Pharmacol, 2013. **84**(3): p. 304-13.
14. Gostissa, M., et al., *IgH class switching exploits a general property of two DNA breaks to be joined in cis over long chromosomal distances.* Proceedings of the National Academy of Sciences of the United States of America, 2014. **111**(7): p. 2644-2649.
15. Schiering, C., et al., *Feedback control of AHR signalling regulates intestinal immunity.* Nature, 2017. **542**(7640): p. 242-245.
16. Diny, N.L., et al., *The aryl hydrocarbon receptor contributes to tissue adaptation of intestinal eosinophils in mice.* Journal of Experimental Medicine, 2022. **219**(4).
17. Smith, B.W., et al., *The aryl hydrocarbon receptor directs hematopoietic progenitor cell expansion and differentiation.* Blood, 2013. **122**(3): p. 376-85.
18. Dong, F., et al., *Intestinal microbiota-derived tryptophan metabolites are predictive of Ah receptor activity.* Gut Microbes, 2020. **12**(1): p. 1-24.
19. Vogel, C.F.A., et al., *Cross-talk between Aryl Hydrocarbon Receptor and the Inflammatory Response A ROLE FOR NUCLEAR FACTOR-kappa B.* Journal of Biological Chemistry, 2014. **289**(3): p. 1866-1875.
20. Ovrevik, J., et al., *AhR and Arnt differentially regulate NF-kappa B signaling and chemokine responses in human bronchial epithelial cells.* Cell Communication and Signaling, 2014. **12**.
21. Meijerink, J., *The Intestinal Fatty Acid-Enteroendocrine Interplay, Emerging Roles for Olfactory Signaling and Serotonin Conjugates.* Molecules, 2021. **26**(5).
22. Munakata, Y., et al., *Olfactory receptors are expressed in pancreatic beta-cells and promote glucose-stimulated insulin secretion.* Scientific Reports, 2018. **8**.
23. Barba-Aliaga, M., P. Alepuz, and J.E. Perez-Ortin, *Eukaryotic RNA Polymerases: The Many Ways to Transcribe a Gene.* Front Mol Biosci, 2021. **8**: p. 663209.
24. Yamada, K., et al., *Nuclear localization of CD26 induced by a humanized monoclonal antibody inhibits tumor cell growth by modulating of POLR2A transcription.* PLoS One, 2013. **8**(4): p. e62304.
25. Ichihara-Tanaka, K., K. Kadomatsu, and S. Kishida, *Temporally and Spatially Regulated Expression of the Linker Histone H1fx During Mouse Development.* Journal of Histochemistry & Cytochemistry, 2017. **65**(9): p. 513-530.
